# Teenage Pregnancy: Obstetric and Perinatal Outcome in a Tertiary Centre in Indonesia

**DOI:** 10.1155/2020/2787602

**Published:** 2020-03-26

**Authors:** Junita Indarti, Adly Nanda Al Fattah, Zulfitri Dewi, Rachmat Dediat Kapnosa Hasani, Fitri Adinda Novianti Mahdi, Raymond Surya

**Affiliations:** Department of Obstetrics and Gynecology, Faculty of Medicine, Universitas Indonesia Cipto Mangunkusumo Hospital, Jakarta, Indonesia

## Abstract

**Background:**

The incidence of teenage pregnancy is increasing in the world. It is a high-risk condition leading to adverse perinatal and obstetric outcomes. This study aims to evaluate the obstetric and perinatal outcomes of teenage pregnancy in Indonesian population.

**Method:**

A retrospective study was conducted to evaluate obstetric and perinatal outcomes among teenagers and average maternal age (AMA) women. We assessed all singleton live pregnancies during the year period of 2013 in Dr. Cipto Mangunkusumo National General Hospital, Jakarta, Indonesia.

**Results:**

We studied 1,676 eligible subjects during the one-year period in our centre. The prevalence of teenage pregnancy (12 to 19 years old) was 11.40% (191/1676). We found higher prevalence of eclampsia (AOR: 4.03; 95% CI: 1.73–9.39), preterm delivery (AOR: 1.5; 95% CI: 0.88–2.53), anaemia at labour (AOR: 2.42; 95% CI: 1.60–3.67), postpartum haemorrhage (AOR: 2.59; 95% CI: 0.86–7.37), and low birth weight (AOR: 2.28; 95% CI: 1.60–3.25) among teenagers. However, caesarean section was found to be significantly lower among teenage pregnancies.

**Conclusion:**

Teenage pregnancy carries significant obstetric complications that should draw physicians' serious attention. A holistic, comprehensive antenatal, and preventive program should be conducted to prevent teenage pregnancy-related adverse outcomes.

## 1. Background

Approximately, 14 million children are born every year to women between 15 and 19 years old [1]. In low- and middle-income countries, nearly 2.5 million births occurred to girls aged under 16 years old [2]. The number is increasing in both well-developed and developing countries [2–5]. It is most likely affected by multifactorial conditions, i.e., socioeconomic problems and low educational level [4, 6, 7]. Teenage pregnancy is considered to be a high-risk condition that leads to psychological problems and adverse perinatal and obstetric outcomes [8–10]. These conditions are not easily solved because they are the result of poor health habit and lack of nutrition [11].

Several studies found various obstetric and perinatal outcomes among teenage pregnancies [10, 12]. Preterm births, preterm premature rupture of membranes, gestational hypertension, preeclampsia, Apgar scores below 7 at the 5^th^ minute, anaemia, poor intrauterine growth, and stillbirths were more prevalent among teenage mothers [10, 13, 14]. In addition, operative vaginal deliveries, caesarean section rate, and low birth rate were significantly higher among women under 19 than those among older ones [15]. Furthermore, there was no greater risk of adverse obstetric outcomes in adolescent women who received adequate prenatal care compared with adult women of similar sociodemographic background [16]. Another study stated that a teenage antenatal clinic would result in better outcomes among teenage pregnancies [17].

Because of the adverse perinatal and maternal outcomes in teenage pregnancy, physicians must pay more attention to and raise their awareness of the management of teenage pregnancy. This study aims to evaluate the obstetric and perinatal characteristics of pregnancies during adolescence in Dr. Cipto Mangunkusumo Hospital, Jakarta, a tertiary university referral hospital in Indonesia.

## 2. Method

We retrospectively assessed women who had singleton live pregnancies and delivered their babies at Dr. Cipto Mangunkusumo Hospital, Jakarta, Indonesia in 2013. These pregnant women were divided into several categories: (1) average maternal age (AMA) women of 20–34 years old; (2) teenagers between 12 and 15 years old; and (3) teenagers between 16 and 19 years old. Preterm birth is defined as delivery before 37 weeks of gestation. The definition of maternal anaemia is based on the criteria set by the Centres for Disease Control (CDC) with a cut-off value of 11 mg/dl for haemoglobin. Preeclampsia is established when the mothers had systolic blood pressure ≥140 mm Hg or diastolic blood pressure ≥90 mm Hg on two occasions 6 hours apart and proteinuria ≥300 mg per 24 hours or +1 using a dipstick test. Severe preeclampsia is said to happen when the mothers have systolic blood pressure >160 mm Hg or diastolic blood pressure >110 mm Hg measured at least twice 6 hours apart, have proteinuria >5 g per 24 hours or ≥+2 using the dipstick test, have eclampsia, have persistent platelet count <100,000/mm^3^, and have serum transaminases of more than twice normal. Eclampsia is established if the preeclampsia patients have a history of seizure. Small-for-gestational age (SGA) is defined as below the 10^th^ percentile for a completed week of gestational age based on the local percentile standards. The percentile of each neonate's birth weight was obtained from the WHO Global Reference for Fetal/Birth weight Percentiles Calculator [18]. Moreover, low birth weight (LBW) is defined as a birth weight of below 2,500 grams. Intrauterine growth restriction is defined as an SGA foetus with Doppler measurement abnormality regarding to obstetricians. Postpartum haemorrhage is defined as bleeding at a rate of more than 500 cc (vaginal delivery) or more than 1,000 cc (caesarean section) or based on the physician's clinical judgement if he or she finds postpartum excessive bleeding that can be caused by either tone, tears, tissue, or thrombin problems. In our institution, induction of labour is usually performed with 25 mcg prostaglandin E1 (PGE1) tablets given 6 hours apart. In this research, oxytocin was given when the ripen cervix had been obtained at a basal infusion rate of 4 mIU/min, and it was increased by 1 mIU/min every 20 minutes until it produced 4-5 uterine contractions every 10 minutes. The maximum applied dose was 20 mIU/min.

Statistical analysis was performed using SPSS 20.0 (IBM Corp. Released 2011. IBM SPSS Statistics for Windows, Version 20.0. Armonk, NY). Kruskal–Wallis test was used for obtaining numerical variables in three groups which were distributed abnormally. The level of significance was set at *p* < 0.05. Multiple logistic regression (the backward method) was performed in order to assess the independent effect of being pregnant in adolescent years on specific indicators of morbidities with significant association.

## 3. Results

There were 1,678 pregnancies from 1 January to 31 December 2013. Of these, 1,487 (88.6%) pregnant women belonged to the AMA group (20–34 years old), 10 (0.6%) pregnant women were 12–15 years old, and 181 (13.1%) were 16–19 years old. Maternal characteristics and neonatal birth weight are described by age groups (Table 1). Incidents of primigravida and anaemia during antenatal care (ANC) were found to be more frequent among teenagers (Table 1). Meanwhile, gestational age at delivery was significantly higher in the AMA women group.

In our study, we observed the association of maternal and neonatal outcomes, such as IUGR, anaemia at labour, preeclampsia, eclampsia, preterm delivery, postpartum haemorrhage, SGA, LBW, APGAR score <7, induction of labour, CS delivery, and instrumental delivery (vacuum or forceps) between two studied groups (Table 2). Those variables were assessed using the Kruskal–Wallis test. Specific indices were significantly different among 12–15-year-old, 16–19-year-old, and 20–34-year-old women. Anaemia at labour, eclampsia, preterm delivery, low birth weight, and CS delivery were statistically more prevalent among teenagers (*p* < 0.001).

Multiple logistic regression was performed to determine the impact of adolescent age on the occurrence of anaemia at labour, eclampsia, preterm delivery, postpartum haemorrhage, LBW, CS delivery, and instrumental delivery. Finally, we observed that, compared with AMA, 12–15-year-old pregnant women had a higher risk of experiencing anaemia at labour (OR: 4.84; 95% CI: 1.22–19.2) and eclampsia (OR: 21.08; 95% CI: 3.89–114.48). Meanwhile, 16–19-year-old pregnant women were more likely to experience anaemia at labour (OR: 1.98; 95% CI: 1.36–2.87), eclampsia (OR: 4.06; 95% CI: 1.74–9.49), and noninstrumental delivery (OR: 2.61; 95% CI: 1.02–6.67), and vaginal delivery (OR: 3.35; 95% CI: 2.29–4.91), compared with AMA women (Table 3).

## 4. Discussion

We found that the percentages of preterm birth, anaemia at labour, eclampsia, postpartum haemorrhage, and LBW were significantly higher among teenagers than those among AMA women. The higher incidence of preterm birth among teenagers in our study was supported by Pergialiotis et al.'s study [10]. Other studies came up with similar findings, which further support the result that young maternal age is a risk factor for preterm births [19–21]. According to the results of a large study, other risk factors such as BMI, employment, previous abortions, previous preterm births, and previous caesarean section contributed to higher incidence of preterm births [22].

A higher incidence of anaemia was significantly seen among teenagers compared with AMA women. Anaemia during delivery was significantly associated with teenage pregnancy. A number of studies showed teenage pregnancy was significantly associated with anaemia [23, 24]. Moreover, Ehrenthal et al. found that women below 20 years old had 1.3 times higher risk for peripartum transfusion, compared with women aged 20–34 years old [25]. In multivariate analysis, anaemia that had been previously detected during the antenatal period was not associated with teenage pregnancy.

Surprisingly, we found a higher risk of eclampsia and postpartum haemorrhage among teenagers in our study. In contrast to our study, a large population study which included 8,514 primiparous women in France found that younger mothers had significantly lower risk of having preeclampsia and postpartum haemorrhage [26]. Complex and numerous factors may play roles in the occurrence of these adverse obstetric or neonatal outcomes. Chen et al. concluded that the increasing risk of neonatal death associated with teenage pregnancy is significantly associated with a higher risk of preterm births. Meanwhile, increasing risk of postneonatal mortality was independently associated with gestational age at birth [27]. To explain the association between teenage pregnancy and adverse obstetric outcomes, we should further study about the quality of antenatal care and socioeconomic factors of the population.

The role of characteristics in teenage pregnancy for analysing maternal adverse outcomes is still ambiguous. Socioeconomic factors such as low education level, being single, and inadequate prenatal care are thought to explain the higher incidence of adverse pregnancy outcomes among teenagers [4, 6, 8]. In addition, poor antenatal care, low nutritional status, and lack of paternal involvement among teenagers were also considered as the risk factors of adverse pregnancy outcomes [3, 28]. However, de Vinne et al. [26] found an association between poor antenatal care and pregnancy outcomes even after the adjustment of socioeconomic characteristics [26].

The strength of our study is a large population observed in a short period of time. We involved more than 1,600 women in a one-year period. To our knowledge, only one study could achieve this number of subjects in a one-year period [10]. A relatively short study period is important because it does not change the protocols in the management of obstetric patients that may potentially affect the maternal and perinatal outcomes. The limitation of our study was its retrospective design. Retrospective design may miss some data in our medical records during analysis. However, our pregnancy reports were directly inputted into our database by competent obstetricians and perinatology residents and checked by their supervisors.

## 5. Conclusion

Teenage pregnancy may cause a significant obstetric complication. In our study, higher prevalence of eclampsia, postpartum haemorrhage, preterm birth, low birth weight, and anaemia was found among pregnant teenagers. However, caesarean section was found to be significantly lower among teenagers. Future studies should evaluate the associated factors explaining a higher incidence of adverse maternal outcomes among teenagers. Therefore, targeted antenatal and preventive programs can be arranged to prevent teenage pregnancies and its concomitant adverse outcomes.

## Figures and Tables

**Table 1:** Maternal characteristics and neonatal birth weight.

Variable	12–15 years old (*n* = 10)	16–19 years old (*n* = 181)	20–34 years old (*n* = 1487)	*p* value
Maternal age (years)^*∗*^	14 (13–15)	19 (16–19)	27 (20–34)	<0.001
Gestational age (weeks)^*∗*^	36 (29–40)	36 (24–41)	38 (23–43)	<0.001
Primigravida, *n* %	9 (90.0)	166 (91.7)	731 (49.2)	<0.001
Birth weight (grams)^*∗*^	2600 (1400–3000)	2500 (800–4200)	2800 (500–4850)	<0.001
Anaemia found during ANC *n* %	4 (44.4)	54 (30.9)	245 (17.2)	<0.001

ANC: antenatal care. ^*∗*^Variables were assessed using the Kruskal–Wallis test and expressed as median (range). All examined variables were statistically significant.

**Table 2:** Maternal and neonatal outcome.

Variable	12–15 years old (*n* = 10)	16–19 years old (*n* = 181)	20–34 years old (*n* = 1487)	*p* value
IUGR	0	5 (2.8)	64 (4.3)	0.496
Anaemia at labour	4 (44.4)	54 (30.9)	245 (17.2)	<0.001
Preeclampsia	1 (10.0)	30 (16.6)	261 (17.6)	0.783
Eclampsia	2 (20.0)	9 (5.0)	30 (2.0)	<0.001
Preterm delivery	6 (60.0)	98 (54.1)	478 (32.1)	<0.001
Postpartum haemorrhage	0	7 (3.9)	22 (1.5)	0.064
SGA	8 (80.0)	158 (87.3)	1294 (87.0)	0.800
LBW	6 (60.0)	92 (50.8)	1061 (71.4)	<0.001
5^th^ min Apgar score < 7	1 (10.0)	7 (3.9)	65 (4.4)	0.647
CS delivery	5 (50.0)	50 (27.6)	702 (47.2)	<0.001
Instrumental delivery (vacuum or forceps)	0	5 (2.8)	100 (6.7)	0.083

IUGR: intrauterine growth restriction; SGA: small-for-gestational age; LBW: low birth weight; CS: caesarean section. ^*∗*^Variables show significant results. All variables were assessed using the Kruskal–Wallis test.

**Table 3:** Multiple logistic regression analysis of obstetric morbidities, neonatal outcome, and mode of delivery.

Variable	12–15 years old OR (95% CI)	*p*	16–19 years old OR (95% CI)	*p*
Preterm delivery	N/A		N/A	
Anaemia at labour	4.84 (1.22–19.20)	0.025	1.98 (1.36–2.87)	<0.001
Anaemia at ANC	N/A		N/A	
Eclampsia	21.08 (3.89–114.48)	<0.001	4.06 (1.74–9.49)	0.001
Postpartum haemorrhage	N/A		2.43 (0.97–6.13)	0.059
LBW	N/A		0.39 (0.28–0.55)	<0.001
Noninstrumental delivery	N/A		2.61 (1.02–6.67)	0.045
Vaginal delivery	N/A		3.35 (2.29–4.91)	<0.001

ANC: antenatal care; LBW: low birth weight; OR: odds ratio; CI: confidence interval; ^*∗*^Variables show significant results.

## Data Availability

The data used to support the findings of this study are available from the corresponding author upon request.
